# Inverse consistent non-rigid image registration based on robust point set matching

**DOI:** 10.1186/1475-925X-13-S2-S2

**Published:** 2014-12-11

**Authors:** Xuan Yang, Jihong Pei, Jingli Shi

**Affiliations:** 1 College of Computer Science and Software Engineering, Shenzhen University, Nanhai Ave 3688, Shenzhen, 518060, China; 2 College of information Engineering, Shenzhen University, Shenzhen, Nanhai Ave 3688, Shenzhen, 518060, China

**Keywords:** Consistent image registration, Robust point matching, Correspondence, Forward transformation, Backward transformation

## Abstract

**Background:**

Robust point matching (RPM) has been extensively used in non-rigid registration of images to robustly register two sets of image points. However, except for the location at control points, RPM cannot estimate the consistent correspondence between two images because RPM is a unidirectional image matching approach. Therefore, it is an important issue to make an improvement in image registration based on RPM.

**Methods:**

In our work, a consistent image registration approach based on the point sets matching is proposed to incorporate the property of inverse consistency and improve registration accuracy. Instead of only estimating the forward transformation between the source point sets and the target point sets in state-of-the-art RPM algorithms, the forward and backward transformations between two point sets are estimated concurrently in our algorithm. The inverse consistency constraints are introduced to the cost function of RPM and the fuzzy correspondences between two point sets are estimated based on both the forward and backward transformations simultaneously. A modified consistent landmark thin-plate spline registration is discussed in detail to find the forward and backward transformations during the optimization of RPM. The similarity of image content is also incorporated into point matching in order to improve image matching.

**Results:**

Synthetic data sets, medical images are employed to demonstrate and validate the performance of our approach. The inverse consistent errors of our algorithm are smaller than RPM. Especially, the topology of transformations is preserved well for our algorithm for the large deformation between point sets. Moreover, the distance errors of our algorithm are similar to that of RPM, and they maintain a downward trend as whole, which demonstrates the convergence of our algorithm. The registration errors for image registrations are evaluated also. Again, our algorithm achieves the lower registration errors in same iteration number. The determinant of the Jacobian matrix of the deformation field is used to analyse the smoothness of the forward and backward transformations. The forward and backward transformations estimated by our algorithm are smooth for small deformation. For registration of lung slices and individual brain slices, large or small determinant of the Jacobian matrix of the deformation fields are observed.

**Conclusions:**

Results indicate the improvement of the proposed algorithm in bi-directional image registration and the decrease of the inverse consistent errors of the forward and the reverse transformations between two images.

## Introduction

Point set matching is a kind of image registration method used widely in the areas of shape matching, motion correction, object recognition and other computer vision applications. The aim of point set matching is to find spatial transformations between two point sets extracted from two images, where the correspondence relationship of points is unknown. Many approaches attempted to solve for point set matching under the affine or projective transformation [1-3]. Recently, there has been considerable interest in point set matching for non-rigid objects [4-10].

The robust point matching (RPM) has become a popular point matching method due to its robustness to disturbances such as noise and outliers. There are two issues needed to be settled for RPM: the correspondence and the transformation. RPM handled these issues generally based on an iterative estimation framework. It utilizes similarity constraints to compute a set of putative correspondences, which include inlier points that there are true correspondence relationship with points in other point set and exclude outlier points without corresponding ones in other point set [5,6,8,9]. And then, under the current estimat of the correspondence, the transformation may be estimated and used to update the correspondence.

Transformations used in RPM can be classified into two categories: non-parametric and parametric. The non-parametric transformation is the one where the geometric deformation is not any parametric mapping functions, such as elastic, fluid and diffusive deformation field. Generally, geometric constraints are needed to estimate the non-parameter transformations. Ma *et al*.[8] used a non-parametric geometrical mapping to formulate the point matching problem as robust vector field interpolation, which took the advantage of regularization the vector field when nonparametric geometric constraint is required. Although point set matching algorithms with non-parametric transformation lead to a globally smooth dense deformation field, they cannot preserve topology of the deformed field.

The parametric transformation is the one where the geometric deformation is represented as parametric mapping functions, such as thin-plate splines (TPS), radial basis function based and affine transformations. Chui *et al*. [4] proposed the TPS-RPM algorithm using TPS to map the source point set to the target point set. Wang *et al*. [7] chose the TPS as the non-rigid deformation function to achieve group-wise registration of a set of shapes represented by unlabelled point-sets. Jian *et al*. [9] employed the TPS and the Gaussian radial basis functions respectively to implement three different cost functions used in RPM. Lian *et al*. [10] applied linear transformation in RPM and reduced the energy function of RPM to a concave function with very few non-rigid terms.

However, whether non-parametric or parametric RPM, the majority of the existing RPM algorithms are asymmetric, that is, the changes measured from transformations are dependent of the order in which the images are registered. When interchanging the order of register images, the RPM algorithm cannot estimate the inverse transformation. Asymmetry problem of registration algorithms lead to biased results when statistical analysis is performed after registration [11]. In order to tackle the asymmetric problem in image registration, symmetric algorithms and inverse consistent algorithms are proposed. Symmetric algorithms optimize cost functions without explicitly penalizing asymmetry. They construct symmetric cost functions by estimating one transformation from one image to another, or construct ordinary cost function by estimating bidirectional transformations to map two images to a common domain using iterative method [12-22]. Bondar *et al*. [12] imposed a symmetry constraint to TPS-RPM by evaluating the correspondence matrix based on the forward and the backward transformations, but the transformation used in TPS-RPM still is unidirectional. Bhagalia *et al*. [16] introduced a bi-directionality term to the RPM objective function, their aim is to reduce the mapping errors in both forward and backward directions for points only, instead of enforcing the forward and the backward transformation to be inverse to each other.

Alternatively, inverse consistent algorithms introduce consistency constraints to the cost function and estimate the forward and backward transformations at the same time [23-32]. Consistency of the forward and backward transformations constrains the forward and backward transformations to be inverses to each other, which ensures that the correspondence produced by the forward transformation is consistent with the correspondence produced by the backward transformation. The idea of inverse consistent image registration is first proposed by Christensen *et al*. [23], in which inverse consistency constraint introduced and added to the matching criteria of images. Johnson *et al*. [24] developed the idea of Christensen and other authors. They proposed the Consistent Landmark Thin-Plate Spline (CLTPS) registration algorithm to estimate the forward and backward transformations between two images based on the correspondence of landmarks. However, the correspondence of control points cannot be ensured during the iterative procedure of the CLTPS algorithm. Furthermore, Christensen *et al*. [25] employed Johnson *et al*.'s algorithm to track lung motion using CT images of multiple breathing periods. He and others [26] concatenated a sequence of small deformation transformations using Johnson *et al*.'s algorithm to estimate the forward and backward large deformation transformations concurrently. Gholipour *et al*. [27] introduced the inverse consistency to a cost function based on a parametric free-form deformation model with a regular grid of control points. Algorithms in [23-27] are based on a parameterized function model. On the other hand, the consistency constraints are also introduced into the registration algorithms based on the dense non-parametric model. Zhang *et al*. [28] employed consistency constraints in a variational framework for multi-modal images registration. Leow *et al*. [29] only solved the forward transformation by directly modelling the backward transformation using the inverse of the forward transformation in unbiased MRI registration. They employed the symmetrizing Kullback-Leibler(KL) distance between the identity map and the transformation, and showed that symmetrizing KL distance is equivalent to considering both the forward and backward transformations in image registration. Tao *et al*. [30] implemented a symmetric and inverse consistent diffeomorphic registration algorithm by avoiding explicit calculation of the inverse deformation. The inverse consistent registration algorithms produce the kind of deformation results that maintain the neighbourhood relationship and present more biological meaning. They produce better correspondence between medical images and smoother displacement fields compared with unidirectional registration algorithms.

The main focus of the paper is to estimate the inverse consistent parametric transformations in RPM. The TPS is the most commonly used parametric transformation in RPM. Although TPS produces a smooth transformation from one image to another, it does not define a consistent correspondence between the two images except at the location of control points [24]. Correspondingly, the transformation solved by the TPS-RPM is unidirectional, that is, the forward and the backward transformations cannot be ensured to be inverted to each other, and the correspondence defined by the forward transformation is different from the correspondence defined by the backward transformation in TPS-RPM.

Presently, to the best of our knowledge, there is no an inverse consistent registration method that can find the forward and backward transformation between two images by matching the sets of points of two images. In this paper, we present an inverse consistent registration algorithm based on robust point matching. The main contributions of this paper as follow. Firstly, we introduce inverse consistency constraint in the RPM cost function, and estimate the forward and backward transformations for two sets of point simultaneously using modified CLTPS. We modify the CLTPS algorithm to improve accuracy of point-to-point mapping in consistent transformations. Secondly, the fuzzy correspondence relationships between points are estimated based on both the forward and backward transformations. Image similarity is also incorporated to the corresponding relationship between points in order to reduce the mismatch of points.

An earlier version of this article was published in the IEEE International Conference on Bioinformatics and Biomedicine (BIBM) hold on 18-21 December 2013 [31] and the sections about consistent robust point matching are from that article. In this paper, we introduce the regularized TPS to preserve the topology of the deformation fields, and estimate the forward and backward transformations during the complete iterative process of point matching, instead of at the end of the iterative process. We further introduce the modified consistent landmark thin-plate spline registration to the complete iterative process of robust point matching. The convergence of our algorithm is demonstrated by experiments. Additionally, we correct the experiment results of RPM in [31] and conduct some new experiments to further compare the performance of inverse consistent RPM using CLTPS in results.

## Methods

### TPS-RPM review

We first review the mathematical framework of TPS-RPM proposed by Chui *et al*. [4]. Given the source point-set *X *= {*x_i_, i *= 1, 2*, . . . , K*} and the target point-set *Y *= {*y_j _, j *= 1, 2*, . . . , N*} in a region Ω, the goal of TPS-RPM is to find the optimal transformation *h *: Ω *→ *Ω that maps the source point-set *X *to the target point-set *Y *, as well as estimating the corresponding relationship between *X *and *Y *. In TPS-RPM, TPS is employed to model the transformation with parameters (*a*, **W**), which maps points in *X *as,

(1) $$h\left(x_{i};a,W\right)=a\cdot x_{i}+\overset{K}{\underset{j=1}{\sum }}w_{j}\cdot \phi \left(r_{ij}\right),$$

where *a *and **W **are affine transform matrix and warp coefficient matrix respectively, *w_j _*is an element of matrix **W**, *r_ij _*= ||*x_i _− x_j_*|| is the distance norm between point *x_i _*and *x_j _, ϕ*(*r_ij_*) is the basis function of TPS.

A fuzzy correspondence matrix *M *with dimension (*K *+ 1) *× *(*N *+ 1) is defined to describe the correspondence between points. Since the one-to-one correspondence relationship between point sets *X *and *Y *will probably not always exist, outlier point is defined as corresponding point of the isolated point. Therefore, each row and each column of matrix *M *has an extra outlier point. The fuzzy correspondence of point *x_i _*and *y_j _*is defined as follows:

(2) $$m_{ij}=\frac{1}{T}e^{\frac{-||y_{j}-h\left(x_{i}\right)||}{2T}},$$

where *T *is the temperature in the anneal procedure of TPS-RPM. The fuzzy correspondence matrix is subject to $\sum_{i=1}^{K+1}m_{ij}=1$ for *j *∈ {1, 2*, . . . , N*}, $\sum_{j=1}^{N+1}m_{ij}=1$ for *i *∈ {1, 2*, . . . , K*}, and *m_ij _*∈ [0, 1]. The nearer the distance between the mapped *x_i _*and *y_j _*is, the more likely a corresponding relationship exists between *x_i _*and *y_j _*.

 RPM employed soft assign and deterministic annealing technique to estimate the fuzzy correspondence matrix *M *and the transformation *h *simultaneously that minimize the following cost function:

(3) $$\begin{array}{cc} E\left(M,a,W\right) & =\overset{K}{\underset{i=1}{\sum }}\overset{N}{\underset{j=1}{\sum }}m_{ij}{∥y_{j}-h\left(x_{i};a,W\right)∥}^{2}+\lambda {∥Lh∥}^{2}\\ & \;\;\;\;\;\;\;\;\;+T\overset{K}{\underset{i=1}{\sum }}\overset{N}{\underset{j=1}{\sum }}m_{ij}\text{log}m_{ij}-\zeta \overset{K}{\underset{i=1}{\sum }}\overset{N}{\underset{j=1}{\sum }}m_{ij}, \end{array}$$

subject to $\sum_{i=1}^{K+1}m_{ij}=1$ for *j *∈ {1, 2*, . . . , N*}, $\sum_{j=1}^{N+1}m_{ij}=1$ for *i *∈ {1, 2*, . . . , K*}, and *m_ij _*∈ [0, 1]. Here, *h *is the transformation maps two point-sets with components *h_d_, d *= 1*, . . . , D*, where *D *is the dimension of Ω. *L *is the linear elastic operator, and ||*Lh*||^2 ^is used to measure the smoothness of the transformation *h*, i.e.

(4) $$||Lh|{|}^{2}=\overset{D}{\underset{d=1\alpha_{1}}{\sum }}\underset{+\cdots +\alpha_{D}=2}{\sum }\frac{2!}{\alpha_{1}!\ldots \alpha_{D}!}\int_{R^{D}}{\left(\frac{\partial^{2}h_{d}}{\partial u_{1}^{\alpha_{1}}\ldots \partial u_{D}^{\alpha_{D}}}\right)}^{2}\underset{j}{\prod }du_{j}$$

The cost function is derived from a statistical physics model. The term $\sum_{i=1}^{K}\sum_{j=1}^{N}m_{ij}\text{log}m_{ij}$ is a barrier function, which is used to push the minimum of the cost function away from the discrete points. The temperature *T *contorls the degree of convexity of the cost function [3]. When *T *is sufficiently small, the cost function is ensured to be convex. *λ *and *ζ *are regularization parameters. In the TPS-RPM algorithm, Expectation-maximization (EM) algorithm is adopted to solve *M *and *h *iteratively, the detailed process can be found in literature [4].

When TPS-RPM is used to register the source image *I *and target image *J *, the source point set *X *and the target point set *Y *are extracted from *I *and *J *respectively. Next, TPS-RPM is employed to estimate the forward transformation *h *: *X → Y *, which is the transformation to map the source image *I *to the target image *J *so that *I*(*h*(*x*)) = *J *. When image *J *is registered to image *I*, the backward transformation *g *: *Y → X* maps the image *J *to image *I *so that *J *(*g*(*x*)) = *I*. As previously mentioned, it is required that the forward transformation and the backward transformation are inversely consistent, i.e. *g ○ h *= *id *and *h ○ g *= *id*, where *id *is the identity map, to ensure the correspondence between the two images to be consistent. However, the forward transformation *h *and the backward transformation *g *is not dependent to each other for TPS-RPM, since TPS is an unidirectional function which results in a non-consistent correspondence between the two images except at the control points, that is, *g ○ h *≠ *id, h ○ g *≠ *id *and *g ○ h *≠ *h ○ g*. Furthermore, the value of the fuzzy correspondence matrix *M *is computed based on the mapping errors in the forward transformation only, the mapping errors from *Y *to *X *will not be penalized, which leads to a bias matching result.

### Inverse consistent robust point matching

Firstly, we introduce several notations used in this paper. The forward transformation from the source point set *X *to the target point set *Y *is denoted as *h*(*x*), the displacement field is *u*(*x*) = *h*(*x*) *− x*; the backward transformation from *Y *to *X *is denoted as *g*(*x*), the displacement field is *w*(*x*) = *g*(*x*) *− x*. The inverse of the forward transformation is *h*^*−*1^, the corresponding displacement field is $\tilde{u}(x)=h^{-1}(x)-x$, and the inverse of the backward transformation is *g*^*−*1^, the corresponding displacement field is $\tilde{w}\left(x\right)=g^{-1}\left(x\right)-x$. An inverse consistent registration is required to satisfy *g ○ h *= *id *and *h ○ g *= *id*. In other words, the forward and backward transformations estimated by an inverse consistent registration should satisfy *g *= *h*^*−*1 ^and *h *= *g*^*−*1 ^in the region Ω. Johnson *et al*. [24] defined the inverse consistency constraint as ||*h − g*^*−*1^||^2 ^+ ||*g − h*^*−*1^||^2^, which makes sure that the function of forward transformation is similar with the inverse function of backward transformation. Correspondingly, the function of backward transformation is as similar with the inverse function of forward transformation as possible. We impose the inverse consistency constraint on the RPM optimization problem by minimizing the cost function given by

(5) $$\begin{array}{cc} E\left(M,a,W\right) & =\overset{K}{\underset{i=1}{\sum }}\overset{N}{\underset{j=1}{\sum }}m_{ij}{∥y_{j}-h\left(x_{i}\right)∥}^{2}+\overset{N}{\underset{j=1}{\sum }}\overset{K}{\underset{i=1}{\sum }}m_{ji}{∥x_{j}-g\left(y_{i}\right)∥}^{2}\\ & \;\;\;\;\;\;\;\;\;+\lambda \left(||Lh|{|}^{2}+||Lg|{|}^{2}\right)+T\overset{K}{\underset{i=1}{\sum }}\overset{N}{\underset{j=1}{\sum }}m_{ij}\text{log}m_{ij}\\ & \;\;\;\;\;\;-\zeta \overset{K}{\underset{i=1}{\sum }}\overset{N}{\underset{j=1}{\sum }}m_{ij}+\chi \left(||h-g^{-1}|{|}^{2}+||g-h^{-1}|{|}^{2}\right). \end{array}$$

In (5), the mapping errors between two point sets are extended as the combination of distance between the target point and the mapped position of the source point using the forward transformation, and the distance between the source point and the mapped position of the target point using the backward transformation, instead of only using the forward mapping errors. Both the smoothness of the forward and backward transformations ||*Lh*||^2 ^+ ||*Lg*||^2 ^are included in the cost function. *Χ *is the weighting parameters to make a trade-off between the inverse consistent error and other terms.

The goal of the inverse consistent robust point matching is to estimate the inversely consistent forward and backward transformations for two sets of points concurrently, as well as making clear the correspondence between *X *and *Y *bidirectionally. The correspondence matrix in traditional RPM is only based on the unidirectional transformation between the target point set and the source point set, while the value of correspondence *m_ij _*for two points in our algorithm is inversely proportional to the mapping errors of points bi-directionally, i.e.

(6) $$m_{ij}=\frac{1}{T}e^{\frac{-\left(\left||y_{j}-h\left(x_{i}\right)||+||x_{i}-g\left(y_{j}\right)|\right|\right)}{2T}}.$$

Furthermore, to register images, the similarity of local image is introduced to the correspondence as,

(7) $$\begin{array}{c} m_{ij}=\frac{1}{T}e^{\frac{-\left(\left||y_{j}-h\left(x_{i}\right)||+||x_{i}-g\left(y_{j}\right)|\right|\right)}{2T}}e^{\frac{-(sim_{h}{\left(J,y_{j},I,x_{i}\right)}^{2}+sim_{g}{\left(I,x_{i},J,y_{j}\right)}^{2})}{2T_{s}}},\\ sim_{h}\left(J,y_{j},I,x_{i}\right)=1-corr\left(J\left(y_{j}\right),I\left(h\left(x_{i}\right)\right)\right),\\ sim_{g}\left(I,x_{i},J,y_{j}\right)=1-corr\left(J\left(g\left(y_{j}\right)\right),I\left(x_{i}\right)\right), \end{array}$$

where, *I*(*x_i_*) and *J *(*y_j _*) are two local regions centred at *x_i _*in image *I *and *y_j _*in image *J . I*(*h*(*x*)) and *J *(*g*(*x*)) are deformed images of *I *and *J *using the forward transformation and the backward transformation respectively. *corr *is the correlation coefficient used to measure the similarity between two local regions. *T_s _*is the temperature parameters of image similarity. By introducing image information to the fuzzy correspondence matrix, improvement of image matching is achieved for the inverse consistent RPM.

To find *M, h *and *g *that used to optimize formula (5), we still use the iterative strategy proposed in [4]. The iterative process includes the **E **step to calculate the fuzzy correspondence matrix according to the current estimated forward and backward transformations. Next, it performs the **M **step to estimate the forward and backward transformations on the basis of the current estimated fuzzy correspondence matrix. By dropping the terms independent of *h *and *g*, it is needed to minimize the following objective function:

(8) $$\begin{array}{cc} E_{c}\left(h,g\right) & =\overset{K}{\underset{i=1}{\sum }}{∥v_{i}-h\left(x_{i}\right)∥}^{2}+\overset{N}{\underset{j=1}{\sum }}{∥z_{j}-g\left(y_{j}\right)∥}^{2}+\lambda \left(||Lh|{|}^{2}+||Lg|{|}^{2}\right)\\ & +\chi \left(||h-g^{-1}|{|}^{2}+||g-h^{-1}|{|}^{2}\right),\\ & \;\;\;\;\;\;\;\;\;\;\;\;\;\;\;\;\;\;\;\;\;\;\;\;\;\;\;\;\;\;\;\;\;\;\;\;\;\;\;\;\;\;\;\;\;\;\;\;\;\;\;\;\;\;\;\;\;\;\;\;\;s.t.\;\;\;\;h\left(x_{i}\right)=v_{i},g\left(y_{j}\right)=z_{j}, \end{array}$$

where $v_{i}=\sum_{j=1}^{N}m_{ij}y_{j}$*, i *= 1, 2*, . . . , K *and $z_{j}=\sum_{i=1}^{K}m_{ij}x_{i}$, *i *= 1, 2*, . . . , N *are the virtual points computed in the forward and backward directions respectively. Moreover, *v_i _*is expected to be corresponding to *x_i_*, and *z_j _*is expected to be corresponding to *y_j _*also. *v_i _*and *z_j _*are held fixed during the procedure of the **M **step. Then, the optimization problem is to find the optimal forward and backward transformations *h *and *g *given four point sets {*x_i_*}, {*y_j_*}, {*v_i_*} and {*z_j_*}, where {*x_i_*} are corresponding to {*v_i_*}, and {*y_j_*} is corresponding to {*z_j_*}. The iterative process continuously alternates the **E **step with the **M **step until it converges. Next, we will discuss how to calculate transformations *h *and *g *at the same time by optimizing *Ec*(*h, g*).

### Modified consistent landmark thin-plate spline registration

Given two point sets with known correspondence relationship, the Consistent Landmark Thin-Plate Spline (CLTPS) registration algorithm [24] was originally proposed to solve the inversely consistent transformations between these two point sets. During the procedure of CLTPS, only two point sets are used to estimate the forward and backward transformations simultaneously. However, there are four point sets {*x_i_*}, {*y_j_*}, {*v_i_*} and {*z_j_*} in RPM. Based on the correspondence between {*x_i_*} and {*v_i_*}, and the correspondence between {*y_j_*} and {*z_j_*}, an intuitive approach to estimate the forward transformation *h *is to let {*x_i_*} be the source point set and {*v_i_*} be the target point set. Conversely, let {*y_j_*} be the source point set and {*z_j_*} be the target point set to estimate the backward transformation *g*. Details of CLTPS can be referred in [24].

However, there several existed problems in CLTPS: (1) the mapped positions of control points are oscillated near their target positions, instead of mapping exactly to the target positions [31]; (2) topology of the forward and backward transformations cannot be ensured to be preserved.

Firstly, there is a minor oscillation problem in CLTPS algorithm. In CLTPS, the forward and backward displacements are updated iteratively using the temporary forward and temporary backward transformations *f*_1_(*x*) and *f*_2_(*x*), where *f*_1_(*x*) is estimated by considering the current mapped position of {*x_i_*} and {*v_i_*} as the source and target control point sets respectively, and *f*_2_(*x*) is estimated by considering the current mapped position of {*y_j_*} and {*z_j_*} as the source and target control point sets respectively. However, in CLPTS, *x_i _*can be mapped to a location near to *v_i_*, but cannot be mapped to *v_i _*exactly. The same goes for *y_j _*also. To tackle the oscillation problem of CLTPS, we propose a new approach to update the forward and backward displacements iteratively. Denote *r_i _*and *s_j _*as the temporary mapped positions of *x_i _*and *y_j _*respectively. After the *k*th iteration, *x_i _*is mapped to *r_i _*using the current forward displacement *u_k_*(*x*), and *y_j _*is mapped to *s_j _*using the current backward displacement *w_k_*(*x*). We update the forward and backward displacements iteratively as follows:

$\begin{array}{c} u_{k+1}\left(x\right)=u_{k}\left(x\right)+\alpha u_{k}^{*}\left(x\right),\;\;\;\;\;\;u_{k}^{*}\left(x\right)=u_{t}\left(u_{k}\left(x\right)+x\right),\;\;\;\;\;\;u_{t}\left(x\right)=f_{1}\left(x\right)-x,\\ w_{k+1}\left(x\right)=w_{k}\left(x\right)+\alpha w_{k}^{*}\left(x\right),\;\;\;\;w_{k}^{*}\left(x\right)=w_{t}\left(w_{k}\left(x\right)+x\right),\;\;\;\;w_{t}\left(x\right)=f_{2}\left(x\right)-x. \end{array}$(9)

We use the forward displacement to demonstrate the improvement of the update. *α *= 1 is assumed so as to simplify the analysis, then, at the *k *+ 1th iteration, the displacement of *x_i _*is,

(10) $$\begin{array}{ccc} u_{k+1}\left(x_{i}\right) & = & u_{k}\left(x_{i}\right)+u_{t}\left(u_{k}\left(x_{i}\right)+x_{i}\right)\\ & = & u_{k}\left(x_{i}\right)+u_{t}\left(r_{i}\right)\\ & = & u_{k}\left(x_{i}\right)+f_{1}\left(r_{i}\right)-r_{i}\\ & = & r_{i}-x_{i}+v_{i}-r_{i}\\ & = & v_{i}-x_{i}. \end{array}$$

It implies that *x_i _*is mapped to *v_i _*exactly using our approach. Similarly, we can prove that *y_j _*is mapped exactly to *z_j _*using the backward displacement.

**Figure 1:**
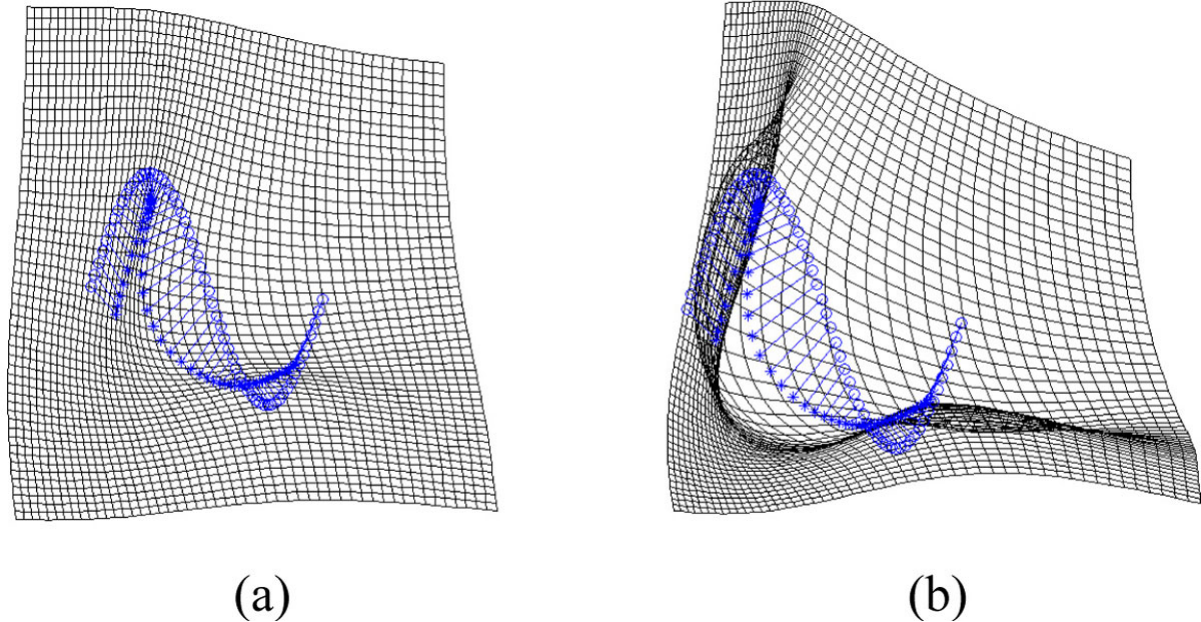
**Grid transformation of TPS and regularized TPS**. (a) is a grid transformation using the regularized TPS. Circle points are source points and star points are target points, the line between two points represents the correspondence of points; (b) is a grid transformation using TPS, it can be seen that folding occurs in the deformation field, which implies that the deformation field is not topology-preserving.

Secondly, the forward and backward transformations estimated by CLTPS cannot be ensured to be topology-preserving, since the temporary transformations *f*_1_(*x*) and *f*_2_(*x*) are estimated by TPS, which does not enforce one-to-one mapping. Topology preservation is an important property of a deformation, which ensures that connected structures remain connected, and that the neighborhood relationships between structures are maintained before and after warping [33]. In image registration, topology preservation of deformation fields can prevent disappearing of existing structures or introducing new artificial structures after image warping. However, transformations estimated by TPS are not constrained to be topology-preserving as they are motivated by small deformation kinematics [34], and they do not allow for large deformations that maintain the topology of the template [35]. To preserve the topology of the deformation field, the regularized TPS proposed by Chui *et al*. [4] is employed to estimate the temporary forward transformation *f*_1_(*x*) and the temporary backward transformation *f*_2_(*x*), which preserves topology of deformation fields better than TPS. As shown in Figure 1, the source points (circle points) are expected to be mapped to the target points (star points). The regularized TPS produces a smooth and topology-preserving deformation field, while TPS makes the deformation field folding, which is non-topology-preserving. The parameter used in the regularization procedure is decreased gradually to preserve the correspondence between points. Moreover, *h *and *g *are required to be topology-preserving, so, after each adjustment of *u*(*x*) and *w*(*x*), the Jacobian values of *h *and *g *are computed, when either of the minimum Jacobian values of *h *and *g *is negative, the iteration is stop.

Finally, *r_i _*and *s_j _*are required to be updated as the newest mapped positions of *x_i _*and *y_j _*for each iteration. So, after the update of the forward and backward transformations, *r_i _*and *s_j _*are updated correspondingly using the latest transformations respectively. More importantly, *r_i _*is closer and closer to *v_i _*with the increase in the number of iterations, rather than swinging nearby *v_i _*as CLTPS. Similarly, *s_j _*is closer and closer to *z_j _*in the iteration process. All these ensures that *x_i _*is mapped exactly to its target position *v_i_*, and *y_j _*is mapped exactly to its target position *z_j _*using the modified consistent landmark thin-plate spline registration algorithm.

Details of the modified consistent landmark thin-plate spline registration are described in algorithm 1.

**Algorithm 1 **Modified Consistent Landmark Thin-Plate Spline (CLTPS) registration algorithm using four points sets.

1: Let *r_i _*= *x_i_, s_j _*= *y_j _*; *u*(*x*) = 0, *w*(*x*) = 0, the steps *α *and *β*, the mapping error threshold *ξ *of control point, and the maximum number of iteration *m_iter_, k *= 1.

2: Regularized TPS is performed to estimate the temporary forward transformation *f*_1_(*x*) based on the correspondence between *r_i _*and *v_i_*, and the temporary backward transformation *f*_2_(*x*) based on the correspondence between *s_j _*and *z_j_*.

3: Update the displacements,

*u*(*x*) = *u*(*x*) + *αu^∗^*(*x*)*, u^∗^*(*x*) = *u_t_*(*u*(*x*) + *x*)*, u_t_*(*x*) = *f*_1_(*x*) *− x*,

*w*(*x*) = *w*(*x*) + *αw^∗^*(*x*)*, w^∗^*(*x*) = *w_t_*(*w*(*x*) + *x*)*, w_t_*(*x*) = *f*_2_(*x*) *− x*.

4: Compute the Jacobian values of the forward and backward transformations, if the minimum Jacobian values of the forward or backward transformations are negative, the iteration is terminated.

5: Get *h*^*−*1^(*x*), the inverse function of forward transformation and *g*^*−*1^(*x*), the inverse function of backward transformation.

6: Update displacement field of forward and backward transformation. *u*(*x*) = *u*(*x*) *− β*[*u*(*x*) *− g*^*−*1^(*x*) + *x*], meanwhile, *w*(*x*) = *w*(*x*) *− β*[*w*(*x*) *− h*^*−*1^(*x*) + *x*].

7: *r_i _*and *s_j _*are updated as *r_i _*= *x_i _*+ *u*(*x_i_*), *s_j _*= *y_j _*+ *w*(*y_j_*).

8: Check whether the termination condition is met. If *k > m_iter _*or *|u*(*x_i_*) *− *(*v_i _− x_i_*)*| < ξ *or *|w*(*y_j_*) *− *(*z_j _− y_j_*)*| < ξ*, the iteration is terminated; otherwise, *k *= *k *+ 1, go to step 2.

## Results

In this section, we will evaluate the performance of inverse consistent RPM algorithm with simulated data and medical images, and also illustrate the efficacy of the image information in estimating the correspondence of points for image registration.

### Synthetic data

**Figure 2:**
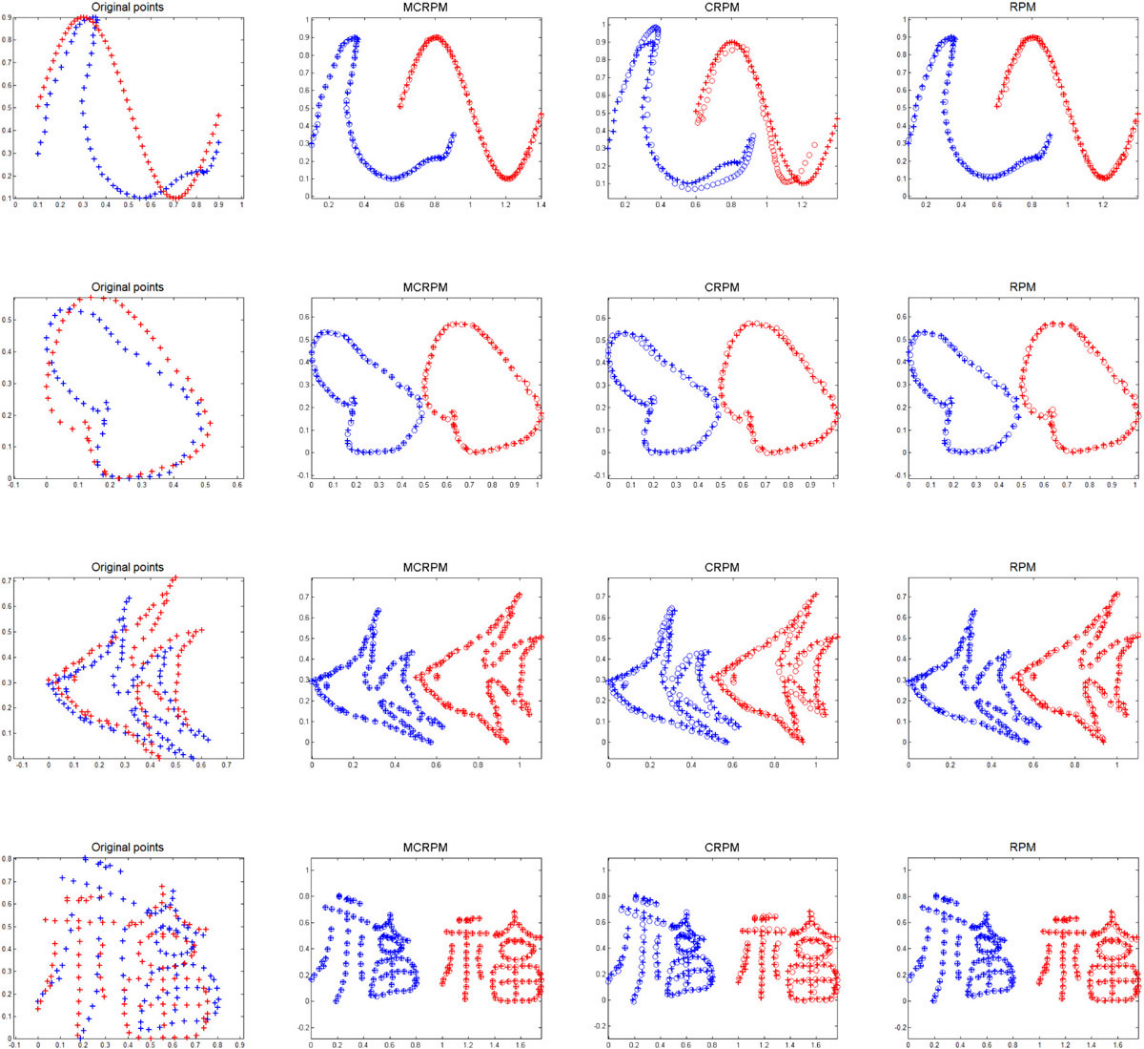
**Registration results of synthetic point sets registration**. From left to right: original points, registered results using MCRPM, CRPM and RPM respectively; In the first column, blue points are target points, red points are source points; In the other three column, blue points are forward registration results, red points are backward registration results, the symbol '+' represents target point sets, the symbol 'o' represents the mapped source point sets.

Four synthetic point sets shown in Figure 2 are used to reveal the performance of the inverse consistent RPM algorithm. We will match the source point set (red pluses) to the target point set (blue pluses). We perform TPS-RPM (RPM), inverse consistent RPM with modified CLTPS (MCRPM), and inverse consistent RPM with CLTPS (CRPM) alternatively.

To determine the behaviours of the forward and backward transformations, a uniform grid in size of 100 *× *100 is employed to be the deformation field of transformations. The inverse consistent error (ICE) of the forward and backward transformations is evaluated by summing the forward consistency error and the backward consistency error, *ICE *= ||*h − g*^*−1*^|| + ||*g − h^−1^*||. Considering that the transformation used in RPM is uni-directional, we perform RPM in the forward and backward directions simultaneously to estimate *h *and *g *respectively. The weighted mapping errors between the target points and mapped source points using the forward transformation, and the source points and mapped target points using the backward transformation are used to define the distance error (DE), $DE=\sum_{i}\sum_{j}m_{ij}\left(\left||y_{j}-h\left(x_{i}\right)||+||x_{i}-g\left(y_{j}\right)|\right|\right)$.

In order to compare the performance of MCRPM, CRPM and RPM, same iteration number is used for three algorithms, the results are shown in Figure 2 (there are some errors in the experiment results of RPM in [31], we correct these errors here). It can be seen that the forward and backward registration results using MCRPM are similar to those using RPM, which implies that the forward registration accuracy of MCRPM is equivalent to that of RPM. Furthermore, both the forward and backward registration accuracy using MCRPM are satisfied. Especially, it is noted that the backward registration error using RPM is not better than that using MCRPM for data 1 and data 2, which demonstrate the advantages of the MCRPM in the bidirectional registration. The bidirectional registration error using CRPM is large for the first and the third point sets, since there is a significant deformation between these two point sets, and the oscillation problem leads the mapped positions of points are not corresponding to their target positions obviously in these cases.

**Figure 3:**
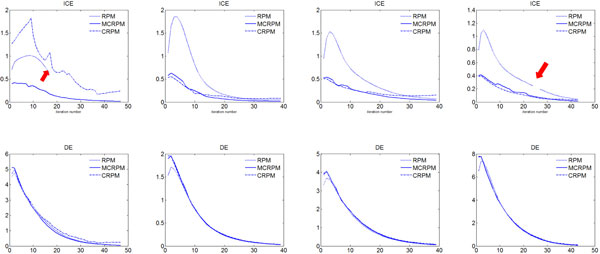
**The inverse consistent error and the distance error for four synthetic point sets**. The red arrows mark the situation of topology non-preservation of transformations. From left to right is data 1 to 4.

Evaluation results are shown in Table 1. It can be seen the inverse consistent errors of MCRPM are smaller than CRPM and RPM. The ICE of CRPM is larger significantly than others for data 1, data 2 and data 3. The reason is that the transformation estimated by CRPM cannot map the source points to the expected positions and vice versa because of the oscillation problem. It further demonstrates the improvement of MCRPM also. Especially, the topology of transformations cannot be preserved for RPM until the end of the iteration for the first data. Figure 3 shows ICE and DE for four data using three algorithms respectively. Noted that the non-topology-preservation of transformation occurs for the first and the fourth data using RPM, which is caused by the large deformation between point sets. The red arrows label the situation of topology non-preservation of transformations. Moreover, the distance errors of MCRPM and CRPM are similar to that of RPM, and they maintain a downward trend as whole, which demonstrates the convergence of MCRPM and CRPM.

**Table 1:** Inverse consistent errors of MCRPM, CRPM and RPM ('-' denotes the topology of transformation is not preserved).

Data	MCRPM	CRPM	RPM
1	0.0150	0.2430	-
2	0.0157	0.0841	0.0423
3	0.0395	0.1538	0.0706
4	0.0137	0.0349	0.0423

**Figure 4:**
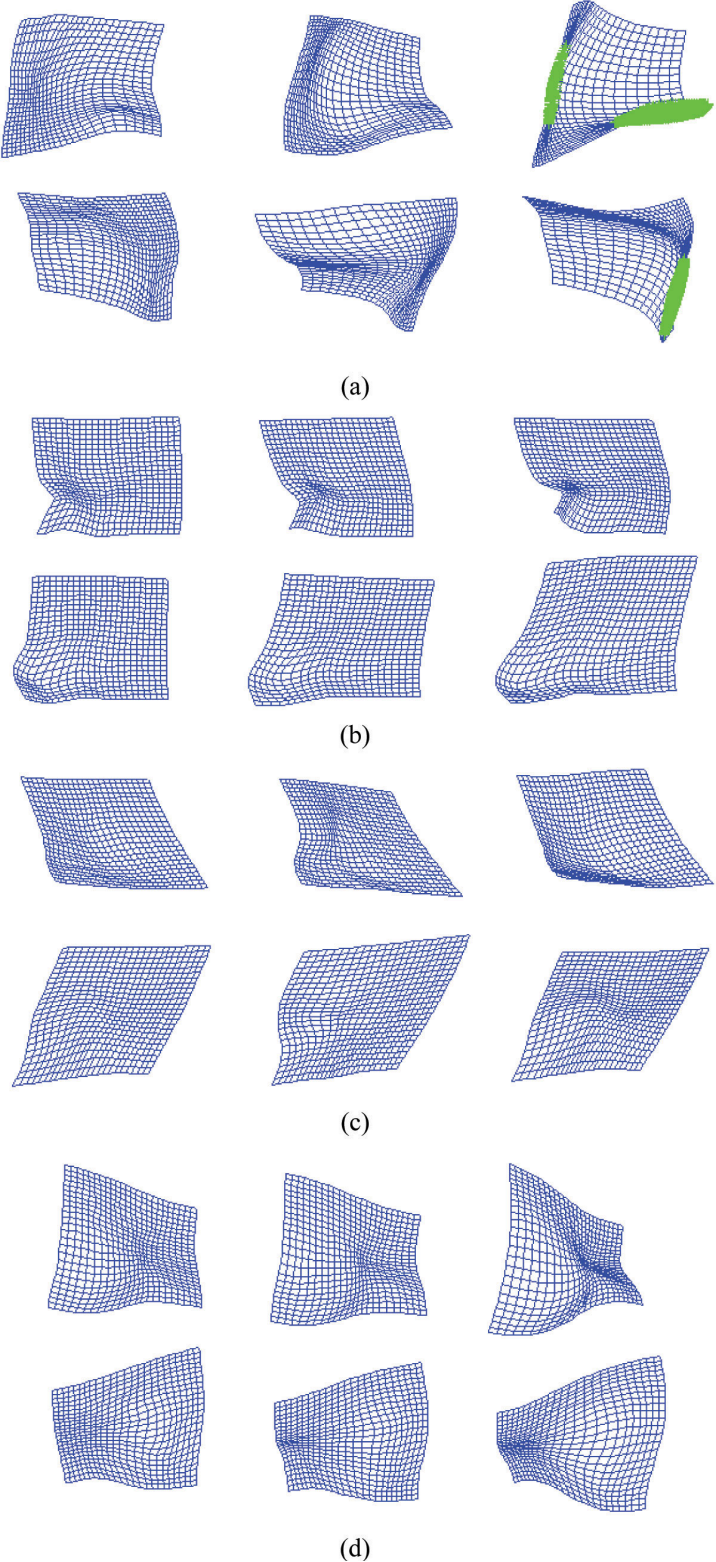
**The deformed fields of four synthetic point sets**. (a)-(d) are the forward and backward deformed fields for data 1 to data 4 respectively. The up rows in (a)-(d) are the forward deformed fields, and the bottom rows in (a)-(d) are the backward deformed fields. From left to right: MCRPM, CRPM and RPM. The area marked by green cross is the topology non-preserving field.

The deformed fields of the forward and backward transformations produced by MCRPM, CRPM and RPM for four synthetic data are shown in Figure 4, respectively. The expanded grids represent expansion of a deformation field, and contracted grids represent contractions of a deformation field. The areas marked by green cross are the topology non-preserving fields. Notice that MCRPM algorithm results in relatively uniform grids in most of the deformed field, compared with that using CRPM and RPM algorithms. Moreover, MCRPM and CRPM have more smooth deformation fields compared with RPM due to the inverse consistent constraints. Especially, the forward and backward transformations produced by RPM cannot preserve topology and lead to artifacts such as "folding" and "tearing" (marked as the green points) of the deformed fields for data 1. These deformed fields show the advantage of using inversely consistent transformations as opposed to using unidirectional transformations.

### Small deformation registration of brain Images

**Figure 5:**
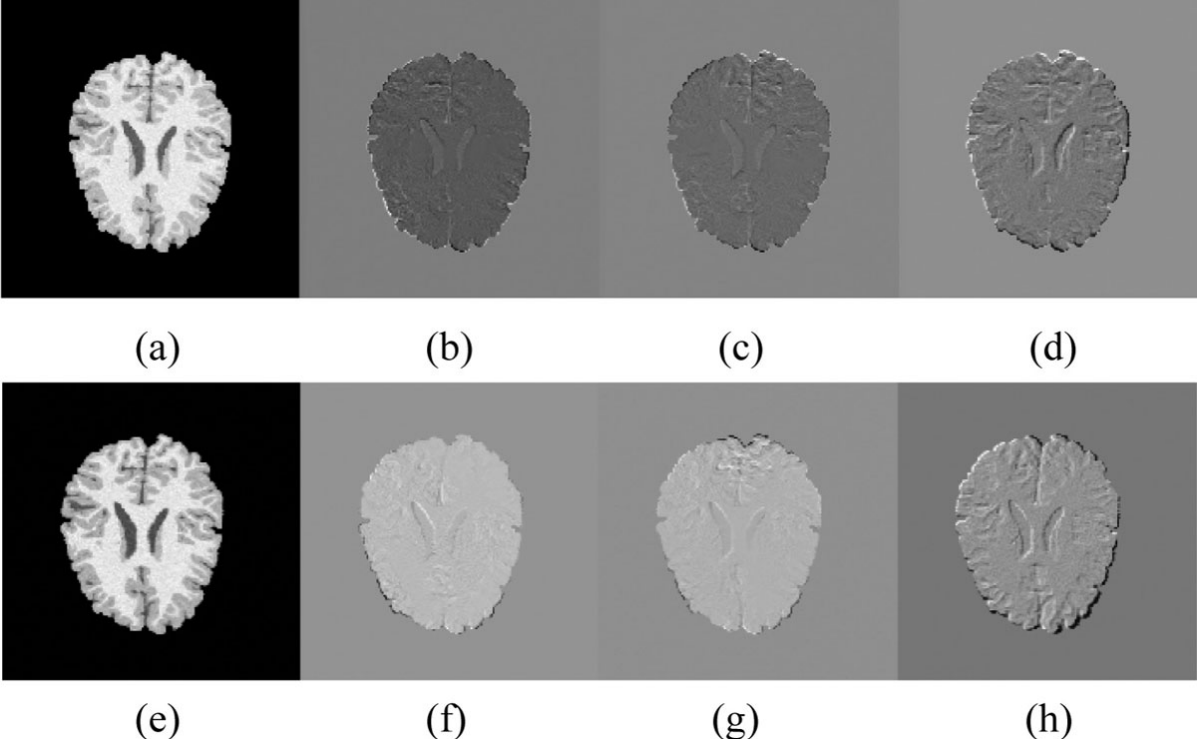
**Image registration results of brain images**. (a):The source image. (b)-(d):The difference between the registered source image and the target image using MCRPM, CRPM and RPM respectively. (e):The target image. (f) - (h): The difference between the registered target image and the source image using MCRPM, CRPM and RPM respectively.

**Figure 6:**
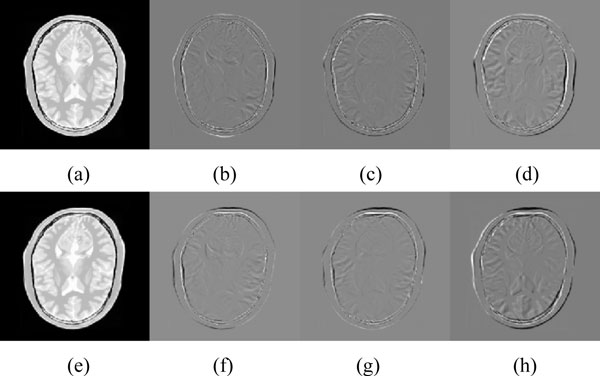
**Image registration results of brain images**. (a):The source image. (b)-(d):The difference between the registered source image and the target image using MCRPM, CRPM and RPM respectively. (e):The target image. (f) - (h): The difference between the registered target image and the source image using MCRPM, CRPM and RPM respectively.

The second example is a consistent image registration, which is used to demonstrate the performance of our approach for registration when the intensity information of images is included. In Figure 5, 6, we show the results of matching two brain images (each of size 256 *× *256). We use two images shown in Figure 5(a) and Figure 6(a) as the target images, and deform the target images manually to get source images, shown in Figure 5(e) and Figure 6(e). These test images are used to evaluate the performance of our algorithm for image registration with small deformation. We will register the source images to the target images, and register the target images to the source images simultaneously. We extract the points from source images and target images respectively, and then perform registration using MCRPM, CRPM and RPM alternatively. The optimal registration results of three algorithms are selected to compare registration accuracy of these algorithms. The mean square deviation (MSD) between the target image and mapped source image using the forward transformation, and the source image and mapped target image using the backward transformation is defined as, $MSD=\sqrt{\frac{1}{N_{x}}\sum_{x}{\left[J\left(x\right)-I\left(h\left(x\right)\right)\right]}^{2}}+\sqrt{\frac{1}{N_{x}}\sum_{x}{\left[I\left(x\right)-J\left(g\left(x\right)\right)\right]}^{2}}$, where *N_x _*is the number of pixels in images.

**Figure 7:**
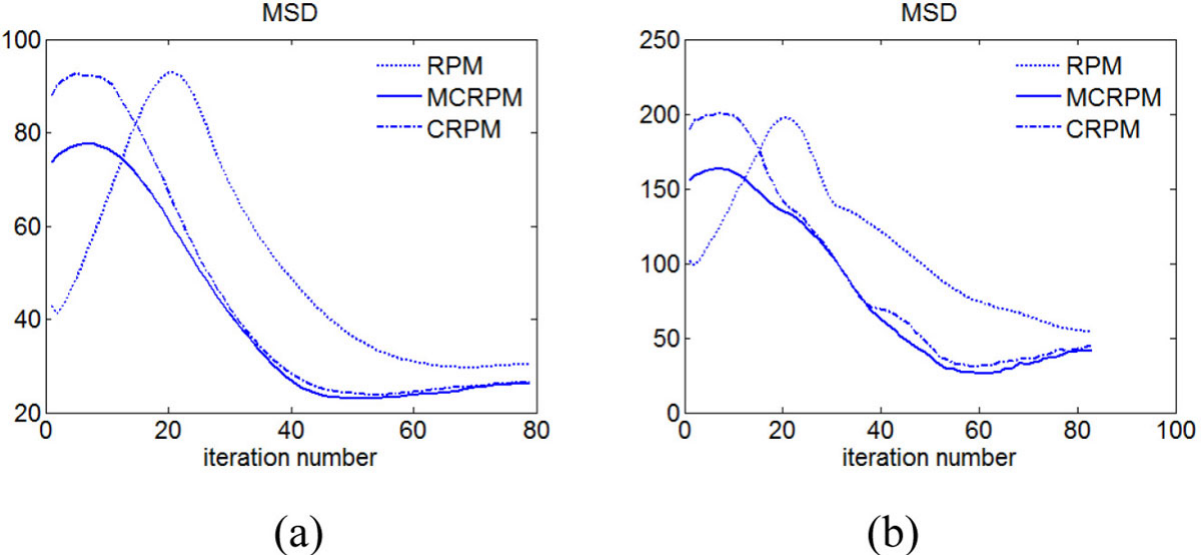
**Accuracy of the forward and the backward image registration**. (a) and (b) shows the mean square deviation of the registration results of Figure 5 and Figure 6 respectively.

Registration results are shown in Figure 5(b)-(d), Figure 5 (f)-(h), Figure 6(b)-(d) and Figure 6 (f)-(h). Here, the parameters *α *= 0.5*, β *= 0.2 are used for the proposed algorithm. It can be seen that RPM is unable to match the inner anatomy of the brain (Figure 5(d) and Figure 5(h)), since the point sets cannot cover the tiny anatomical structure totally and regions without control points are deformed unmanageably using the unidirectional transformation estimated by RPM. It is noted that MCRPM and CRPM register the tiny anatomy of the brain better due to the inversely consistent transformations. Moreover, MCRPM and CRPM matches the outside contour of the brain better than RPM slightly, since RPM does not considering image information similarity between points, which is in the most obvious in matching the outside contours. The MSD for these two image registrations by the three algorithms are shown in Figure 7 respectively. Again, MCRPM achieves the lower registration errors in same iteration number. Also, it is noted that the MSD for registration results using MCRPM and CRPM decreases significantly, which is caused by introducing image information in the estimation of point correspondences.

MSD and inverse consistent error of the optimal registered results using MCRPM, CRPM and RPM are listed in Table 2. It can be seen that MCRPM achieved the optimal results in respect to both the MSD and the inverse consistent error. It is observed that the inverse consistent error using MCRPM and CRPM are better than that using RPM significantly, which demonstrate the advantages of inverse consistent transformations used in the image registration.

**Table 2:** The mean square deviation and mean inverse consistent error of registration results of Figure 5 and Figure 6.

	MSD			ICE		
	
	MCRPM	CRPM	RPM	MCRPM	CRPM	RPM
Figure 5	22.98	23.76	29.63	0.0092	0.0107	0.0242
Figure 6	26.23	31.01	54.13	0.0054	0.0074	0.0233

To further analyse the smoothness of the forward and backward transformations, it is needed to examine the determinant of the Jacobian matrix of the deformation field. The determinates of the Jacobian matrix of *h *and the Jacobian matrix of *g *are denoted as *Det*(*h*) and *Det*(*g*) respectively. The determinant of the Jacobian matrix close to 1 indicates less expansion and contractions at a pixel, which means the deformation at the point is less; the more pixels whose Jacobian determinant values are close to 1 are, the less the deformation field is. To quantify the distance between the deformation and the identity map, *|Det*(*h*) *− *1*| *and *|Det*(*g*) *− *1*| *are calculated and listed in Table 3. Noted that for the small deformation (Figure 5 and Figure 6), the mean values of *|Det*(*h*) *− *1*| *and *|Det*(*g*) *− *1*| *for MCRPM and CRPM are less than that for RPM, which means the forward and backward transformations estimated by MCRPM and CRPM are smooth for small deformation.

**Table 3:** The Jacobian values of the forward and backward transformations of Figure5 and Figure 6.

	|*Det*(*h*) *− *1|			|*Det*(*g*) *− *1|		
	
	MCRPM	CRPM	RPM	MCRPM	CRPM	RPM
Figure 5	0.0098	0.0150	0.0342	0.0201	0.0167	0.0365
Figure 6	0.0163	0.0236	0.0920	0.0198	0.0264	0.0871

### Lung Slices

**Figure 8:**
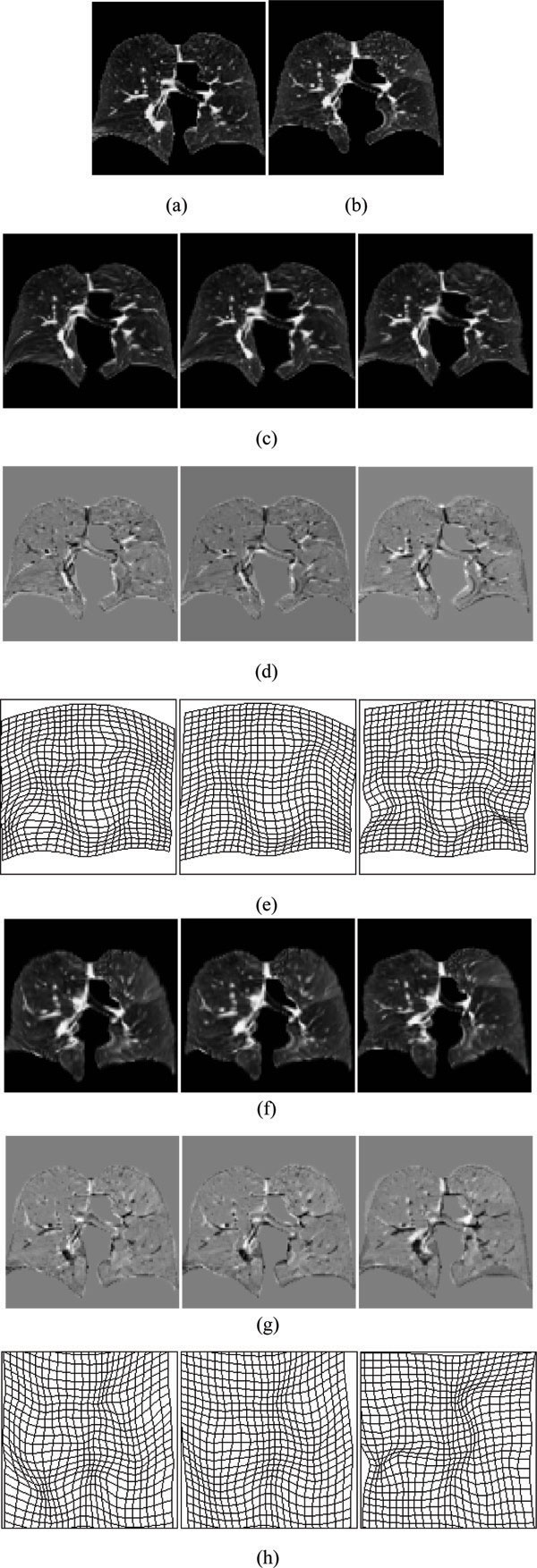
**Registration of lung slices**. (a) and (b) are the source and target images, they are slices with maximum inhale and maximum exhale phases respectively. (c) is the forward deformed results by MCRPM, CRPM and RPM respectively. Corresponding to (c), (d) shows the difference between the source image and the forward deformed results, and (e) is the forward grid transformation. (f) is the backward deformed results by MCRPM, CRPM and RPM respectively. Corresponding to (f), (g) shows the difference between the target image and the backward deformed results, and (h) is the backward grid transformation.

We evaluate the accuracy of registration on thoracic images, which are provided by DIR-lab (http://www.DIR-lab.com) and consist of 10 cases, each one having a thoracic image at with six phases. We extract a slice from the image with the maximum inhale phase as the source image, and the corresponding slice with the maximum exhale phase as the target image. We utilize the slices extracted from 10 cases of thoracic images from DIR-lab to compare registration results of different algorithms as demonstrated in Figure 8. The source and target image are shown in Figure 8(a) and (b), they are slices of a lung with the maximum inhale phase and the maximum exhale phase. Bronchi in the source image might not exist in the target image and vice versa because of the respiratory motion. Correspondingly, there are many outliers in both point sets extracted from the source image and the target image. This experiment is used to demonstrate the performance of inverse consistent registration when many outliers included in the point sets. Images in Figure 8(c) show the forward registered results by MCRPM, CRPM and RPM respectively. Figure 8 (f) shows the registered images in the backward direction by three algorithms, respectively. Noted in both directions, the registered results by MCRPM and CRPM match their template images better than RPM. Especially, the improvement of the registration accuracy at the contours of the lung images can be observed in Figure 8 (d) and (g) also. It demonstrates that our algorithm performs better than RPM when many outliers exist in both point sets simultaneously, because the inherent structure of RPM algorithm does not efficiently handle outliers in this case [5]. Figure 8 (e) and (h) are the forward and backward grid transformations by three algorithms. It can be seen that the transformations estimated by MCRPM and CRPM are smoother than by RPM.

**Figure 9:**
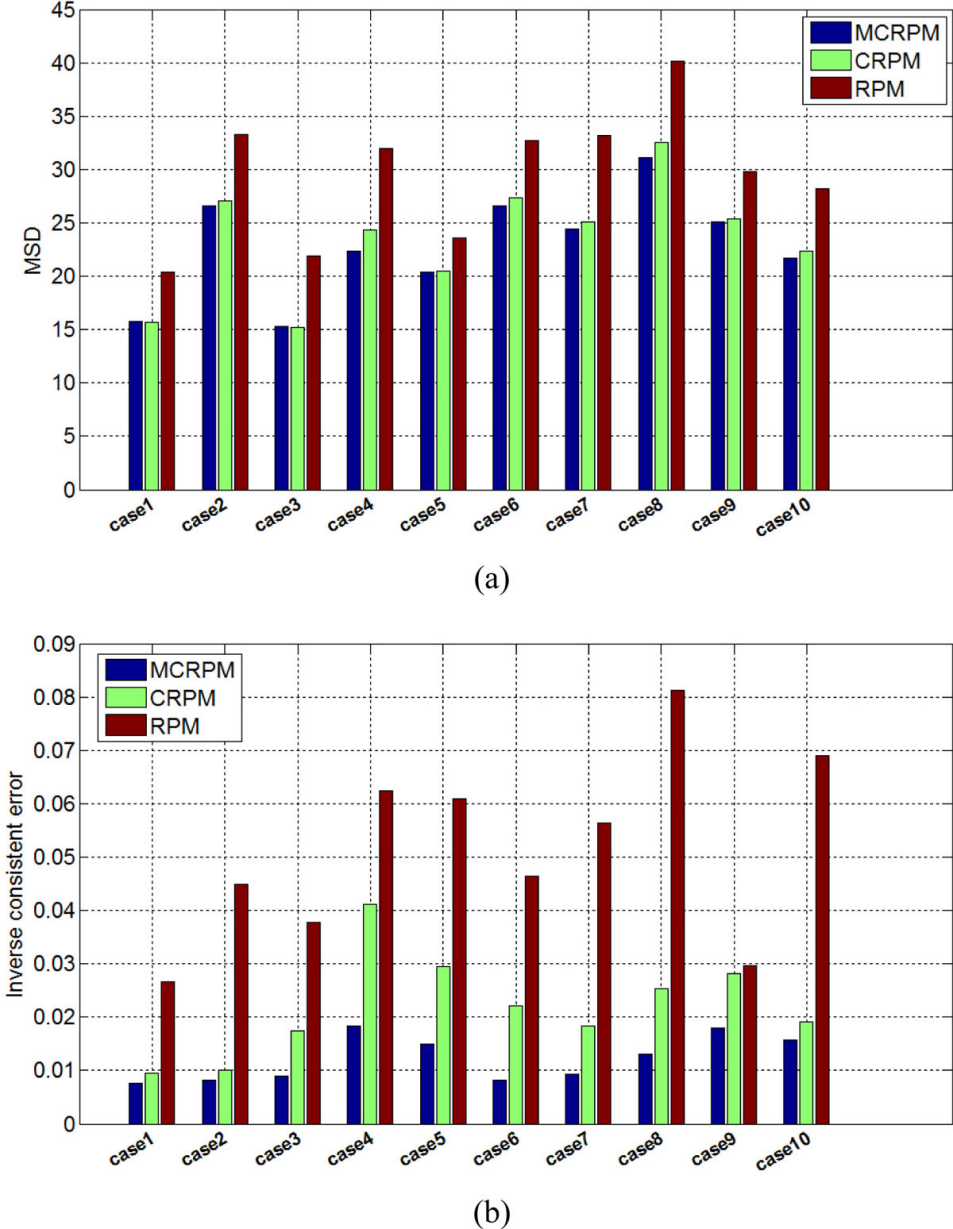
**Lung slice registered results**. (a) and (b) show the MSD and the inverse consistent errors for ten lung slices registration using the MCRPM, CRPM, and RPM respectively.

To illustrate the registration accuracy of all ten cases, Figure 9(a) shows the MSD of registration results using three algorithms respectively. The MSD measure of ten cases illustrates that MCRPM and CRPM achieve the lower registration errors. It is noted that the registration errors of MCRPM are less than that of CRPM, which is due to the improvement of mapping accuracy of points. The inverse consistent errors of ten registration results shown in Figure 9(b) show that whether using the MCRPM or the CRPM, the inverse consistent errors are smaller than that using RPM. Furthermore, MCRPM is better than CRPM in aspect of inverse consistent error also.

Table 4 lists the Jacobian value of the forward and backward transformations for registration of lung slices. It is observed that the mean values of *|Det*(*h*) *− *1*| *and *|Det*(*g*) *− *1*| *for MCRPM and CRPM are larger than that for RPM for many cases. The reason is that the deformations of lung slices registration are non-rigid, it requires large expansion or contraction deformations to match each other. So it is reasonable that large or small values of *Det*(*h*) and *Det*(*g*) are observed.

**Table 4:** The Jacobian values of the forward and backward transformations.

	|*Det*(*h*) *− *1|			|*Det*(*g*) *− *1|		
	
Case	MCRPM	CRPM	RPM	MCRPM	CRPM	RPM
1	0.1159	0.0986	0.1275	0.1324	0.0991	0.1357
2	0.1104	0.0903	0.1026	0.1186	0.0906	0.0896
3	0.1336	0.1435	0.1553	0.1544	0.1639	0.1540
4	0.2086	0.2157	0.2593	0.2351	0.2349	0.2028
5	0.1640	0.1338	0.2396	0.1713	0.1316	0.2863
6	0.1492	0.1716	0.1947	0.1709	0.1771	0.1494
7	0.1034	0.1185	0.1467	0.1054	0.1137	0.0711
8	0.2659	0.2083	0.2259	0.3125	0.2356	0.2913
9	0.0997	0.1125	0.0791	0.1096	0.1261	0.0808
10	0.1445	0.1332	0.2197	0.1290	0.1065	0.1433

### Individual brain images

The fourth experiment contains the same slices extracted from 10 subjects of Brain Web. This experiment is used to demonstrate the performance of our approach for inter-subject image registration when the deformations of images are large. One subject serves as the target image and another image is aligned to the target image. Registration described as subject 1-2 means that subject 1 and subject 2 are used for evaluation.

**Figure 10:**
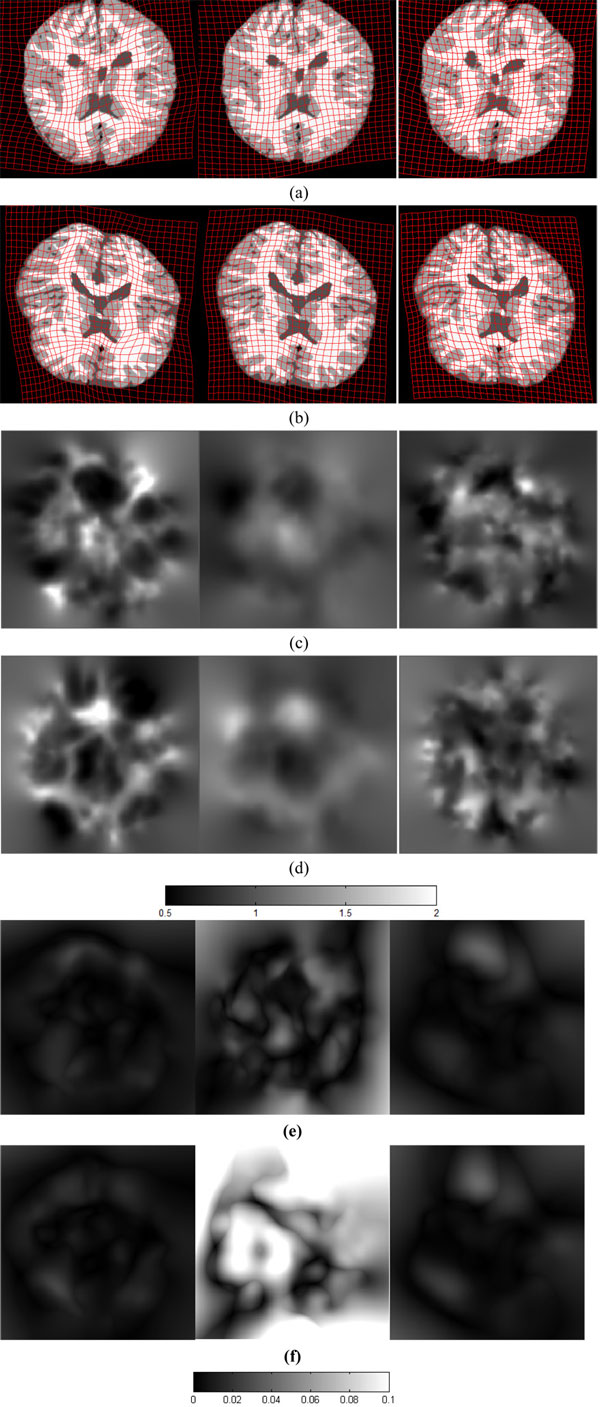
**Individual brain slice registration example**. (a) and (b) show the forward and backward registered results for subject 5 and 6 using the MCRPM, CRPM, and RPM. From left to right: MCRPM, CRPM and RPM. (c) and (d) show the visualization of the Jacobian maps of the forward and backward deformations corresponding to (a) and (b). The bottom two rows (e) and (f) show the intensity pattern of the magnitude and location of the forward and backward transformation inverse consistent errors using three algorithms respectively.

**Figure 11:**
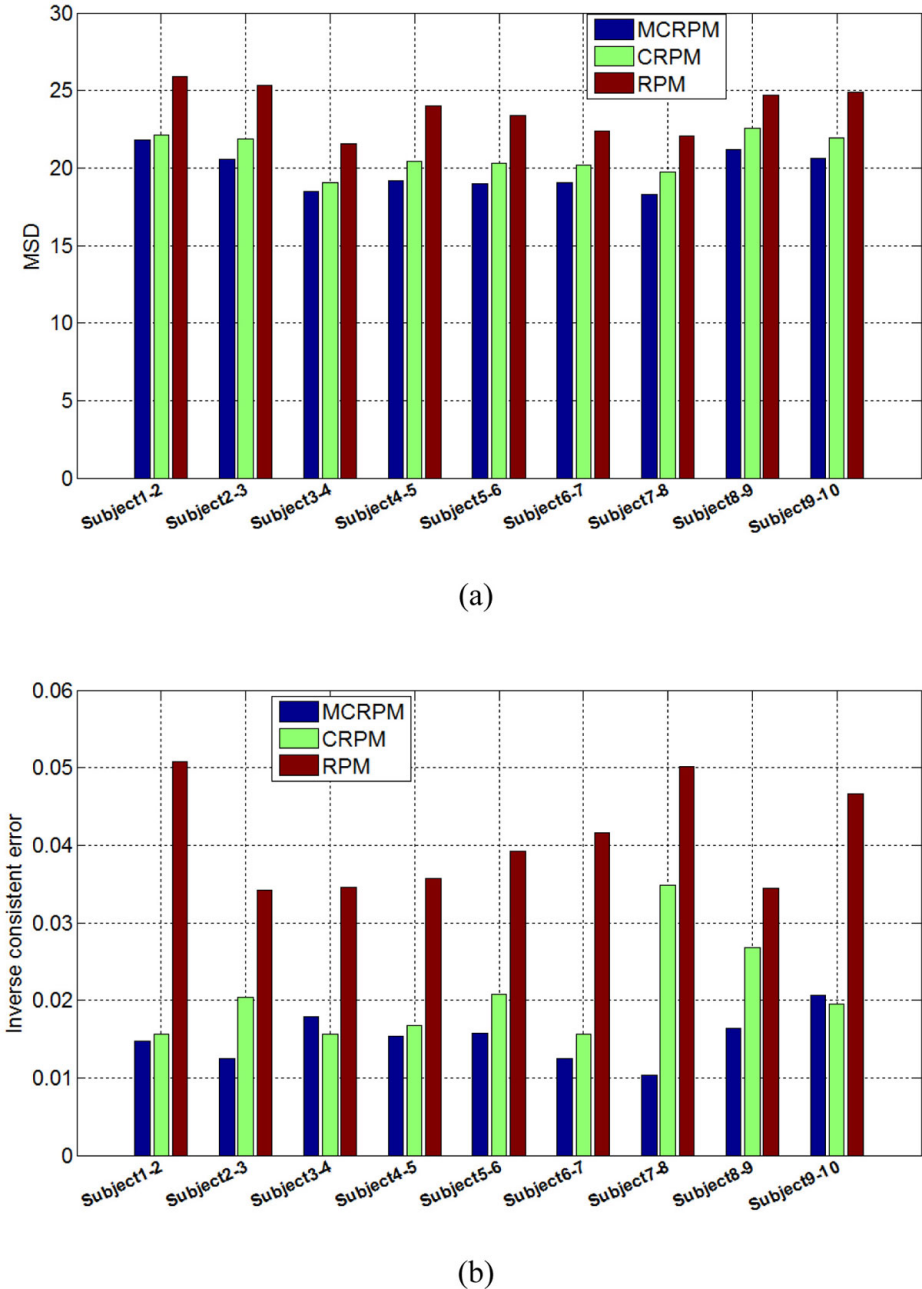
**Individual brain slice registered results**. The first row (a) and the second row (b) show the MSD and the average inverse consistent error for nine individual brain slices registration using the MCRPM, CRPM, and RPM respectively.

To illustrate the proposed algorithm, we demonstrate the registration results of subject5-6, where visually significant deformation is present. As seen in Figure 10(a) and (b), both the MCRPM and CRPM produces a close match between the source image and the target image. However, as seen in Figure 10(b), the RPM deforms the target image to the source image by the backward transformation that is similar to an affine transformation. The reason is that RPM provides more freedom for the affine transformation to avoid unphysical reflection mappings [4]. If this constraint is not introduced, RPM will lead to transformations with large bending energy and result in worse registration results. It exactly demonstrates the advantage of introducing the inverse consistent transformations to non-rigid image registration. Figure 10(c) and (d) illustrates that the Jacobian fields of the forward and backward transformations estimated by three algorithms. It is noted that the intensity pattern of the forward and backward Jacobian fields of the MCRPM and CRPM are closely opposite of one another since MCRPM and CRPM produced inversely consistent transformations, while the similar results cannot be observed in the forward and backward Jacobian fields of RPM. The intensity pattern of the inverse consistent errors of the forward and backward transformations are shown in Figure 10(e) and (f) respectively. Obviously, the inverse consistent errors of MCRPM and CRPM are smaller than that of RPM at almost every pixel location in the image domain. Note that there are large regions of bright pixels in the backward deformation field of RPM, which implies large inverse consistent errors occur in the backward transformation. MSD and ICE measures of nine registered results are shown in Figure 11. Again, MCRPM achieves the lest registration error, and MCRPM and CRPM are better than RPM in respect of inverse consistent error.

**Figure 12:**
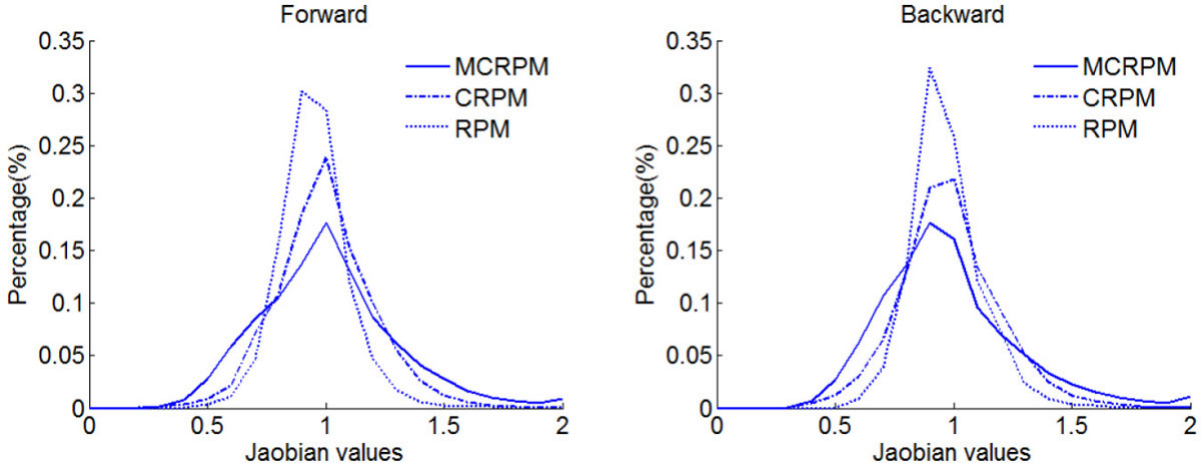
**The Jacobian values histograms of the forward and the backward transformations for individual brain slices registration**.

Furthermore, by collecting the Jacobian values from all pixels, Figure 12 shows the histogram of *Det*(*h*) and *Det*(*g*). As Figure 12 shows, the peek position of Jacobian histogram by the RPM indicates that the deformation by RPM is mainly determined by the affine transformation and the non-rigid deformation is weak. For the problem of inter-subject registration, the deformation is mainly determined by non-rigid transformation rather than the affine transformation, so it indicates that the registration result by the RPM is not satisfied. The distribution of Jacobian value implies that the transformations for MCRPM and CRPM are non-rigid deformation mainly, which is in accord with the deformation of inter-subject registration.

## Conclusions

We proposed a consistent image registration approach by combining the RPM algorithm and modified consistent landmark thin-plate spline algorithm together. It introduced the forward and the backward transformations to the cost function of points matching, and estimated the correspondence matrix based not only on bi-directional transformations but also on the correlation of image content. The forward and backward transformations were estimated during the complete iterative process of point matching. The regularized TPS was introudced to our algorithm to produce topology-preserving transformations for image registration with large deformation, and produce smooth transformations for image registration with small deformation. The modified consistent landmark thin-plate spline algorithm improved the correspondence between points, and significantly reduced the inverse consistent error between the forward and backward transformations. Experiment results demonstrated the convergence of our algorithm, and medical images registration results showed that our algorithm was superior to RPM in aspect of intensity matching between images. A desired improvement in our approach would be to reduce computational time to estimate the inversely consistent transformations.

## Competing interests

The authors declare that they have no competing interests.

## Authors' contributions

XY, JHP & JLS participated in literature search, data analysis, manuscript writing and editing. All the authors read and approved the final manuscript.

## Declarations

Publication of this article has been funded by National Natural Science Foundation of China (U1301251) and Shenzhen Science and Technology Projection (JCYJ20130326112132687).

This article has been published as part of *BioMedical Engineering OnLine *Volume 13 Supplement 2, 2014: Selected articles from the IEEE International Conference on Bioinformatics and Biomedicine (BIBM 2013): BioMedical Engineering OnLine. The full contents of the supplement are available online at http://www.biomedical-engineering-online.com/supplements/13/S2.
